# Treatment as the Best Prevention: Twice-Yearly Lenacapavir, a Game Changer in Ending the AIDS Epidemic

**DOI:** 10.3390/v16091368

**Published:** 2024-08-28

**Authors:** Ziyu Wen, Minjuan Shi, Caijun Sun

**Affiliations:** 1 School of Public Health (Shenzhen), Sun Yat-sen University, Shenzhen 518107, China; wenzy8@mail.sysu.edu.cn (Z.W.); shimj8@mail2.sysu.edu.cn (M.S.); 2 Shenzhen Key Laboratory of Pathogenic Microbes and Biosafety, Shenzhen Campus of Sun Yat-sen University, Shenzhen 518107, China; 3 Key Laboratory of Tropical Disease Control (Sun Yat-sen University), Ministry of Education, Guangzhou 510080, China; 4 State Key Laboratory of Anti-Infective Drug Discovery and Development, School of Pharmaceutical Sciences, Sun Yat-sen University, Guangzhou 510006, China

Despite over four decades of unremitting efforts since the discovery of acquired immunodeficiency syndrome (AIDS)/human immunodeficiency virus (HIV), there remains no cure for HIV nor a vaccine for its prevention. Unsurprisingly, the HIV epidemic continues to devastate communities worldwide, with approximately 39.9 million people living with HIV (PLWH) by the end of 2023. However, the advent and refinement of antiretroviral therapy (ART) have transformed AIDS from a rapidly fatal illness into a manageable chronic disease. Herein, we summarize the winding journey in HIV prevention and control (Figure 1).

The proof-of-concept for the pre-exposure prophylaxis (PrEP) strategy emerged in the mid-2000s with the goal of preventing HIV acquisition among high-risk, HIV-negative individuals through regular use of antiretroviral medications. Currently, more than 144 countries have adopted the World Health Organization (WHO)’s recommendation in their national guidelines to prevent the spread of AIDS. The first generation of PrEP drugs primarily included oral nucleoside reverse transcriptase inhibitors (NRTIs) such as Tenofovir/Emtricitabine (TDF/FTC) and Tenofovir Alafenamide/Emtricitabine (TAF/FTC). Numerous trials have confirmed the efficacy and safety of the PrEP strategy. In 2012, WHO recommended daily oral TDF/FTC as a PrEP intervention to prevent HIV infections among HIV-negative partners in serodiscordant couples, marking a pivotal advancement in HIV prevention [1]. In 2015, this recommendation was extended to include men who have sex with men (MSM) and individuals who inject drugs, heralding a new era in HIV prevention efforts [2]. However, challenges emerged with poor adherence to daily medication observed in VOICE and KPNC trials, significantly weakening the effectiveness of first-generation PrEP drugs [3,4]. Consequently, second-generation PrEP drugs with longer half-lives have been developed to improve adherence. For instance, the dapivirine vaginal ring, a non-nucleoside reverse transcriptase inhibitor (NNRTI) administered monthly, substantially reduced the risk of HIV infection among African women. In 2021, WHO endorsed the dapivirine ring as an additional prevention option for women at high risk of HIV. Cabotegravir, an integrase strand transfer inhibitor administered via bi-monthly injections, demonstrated superior efficacy compared to daily oral TDF/FTC in HPTN 083 and HPTN 084 trials among MSM and transgender women [5]. As a result, WHO recommended long-acting injectable cabotegravir (CAB-LA) as an additional HIV prevention option in 2022 for high-risk individuals. Islatravir (ISL), a novel nucleoside reverse transcriptase translocation inhibitor, is currently under evaluation in the Impower 022 and Impower 024 trials, which are assessing its efficacy among different population. Intravenous infusion of the broadly neutralizing antibodies (VRC01) every 8 weeks also showed prevention efficacy in the HVTN704/HPTN085 (in high-risk women in sub-Saharan Africa) and HVTN703/HPTN081 (in high-risk men and transgender individuals in the Americas and Europe) trials. Lenacapavir, a long-acting capsid inhibitor, has demonstrated potent antiviral activity in PLWH. A single subcutaneous injection of lenacapavir provided sufficient pharmacokinetic exposure for up to six months, and it received approval for HIV prevention in the European Union in 2022 [6].

Notably, Gilead Sciences recently reported their groundbreaking results of the double-blinded, multicenter, randomized Phase III clinical study (PURPOSE 1 trial), demonstrating that twice-yearly subcutaneous administration of lenacapavir achieved 100% efficacy in preventing HIV infections [7,8]. This trial involved 5338 adolescent girls who aged 16–25 across 25 sites in South Africa and 3 sites in Uganda. The results showed zero case of HIV infection among 2134 women in the lenacapavir group (an incidence rate of 0.00/100 person-years). In contrast, there were 16 HIV cases among 1068 women in the Truvada group (once-daily emtricitabine/tenofovir disoproxil fumarate; incidence rate of 1.69/100 person-years) and 39 HIV cases among 2136 women in the Descovy group (once-daily emtricitabine/tenofovir alafenamide; incidence rate of 2.02/100 person-years).

As the first Phase III trial in the history of HIV prevention to show zero infections, PURPOSE 1 may reignite hope for ending the HIV epidemic. The long-acting lenacapavir significantly reduces the frequency of medication, which can greatly improve adherence among individuals who face challenges such as time constraints, privacy concerns, and social stigma associated with daily medication regimens. While the results of the PURPOSE 1 trial are promising, the study only included high-risk young women in Africa, making it necessary to further validate and promotes its efficacy and safety across other populations. Variations in gender, age, and geographic region may lead to different responses to PrEP drugs. Additionally, the current focus of PrEP strategies on high-risk populations leaves gaps in understanding its safety and acceptance among the general population, posing a challenge for AIDS prevention and control efforts. From this perspective, developing an ideal HIV preventive vaccine for the general population remains another critical goal in ending the AIDS epidemic [9,10]. Moreover, increasing public awareness of PrEP and addressing issues of access and affordability are also essential for its widespread clinical application. Overall, in pursuit of the goal of eradicating AIDS, comprehensive strategies including public health education, behavioral interventions, PrEP and post-exposure prophylaxis (PEP), preventive vaccines, and curable drugs must be continually developed and optimized for HIV prevention and control. Only then can we envision a world without AIDS.

## Figures and Tables

**Figure 1:**
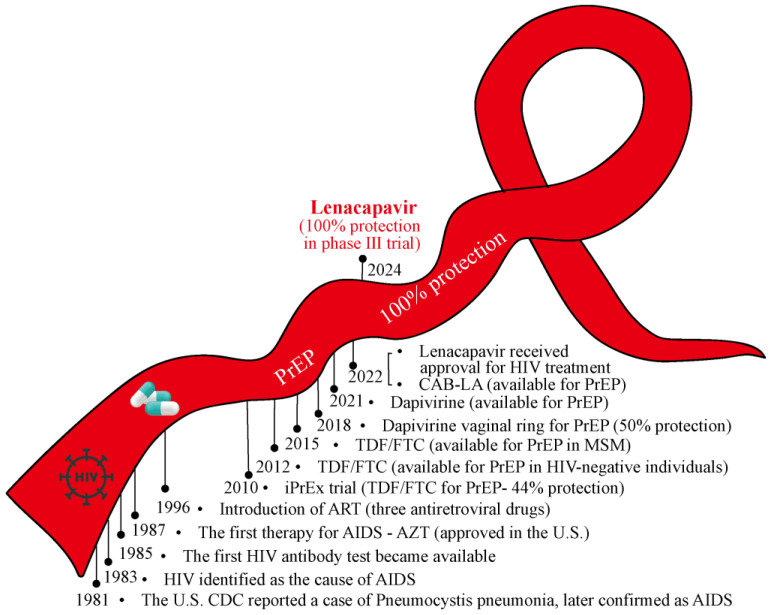
The winding road of the red ribbon in HIV prevention and control. From initial behavioral interventions to advancements in HIV vaccines, PEP, and PrEP, the journey of HIV prevention has been fraught with challenges. The availability of TDF/FTC represents a significant milestone in improving PrEP accessibility. Most recently, the phase III trial of lenacapavir as a PrEP medication achieved 100% efficacy in HIV prevention, offering renewed hope for ending the HIV epidemic. The winding red ribbon symbolizes the ongoing efforts and achievements in the battle against HIV/AIDS. ART: Antiretroviral Therapy; PEP: Post-exposure Prophylaxis; PrEP: Pre-exposure Prophylaxis; AZT: Azidothymidine; TDF: Tenofovir; FTC: Emtricitabine; CAB-LA: Long-acting Injectable Cabotegravir.
